# Continuous Modeling of Arterial Platelet Thrombus Formation Using a Spatial Adsorption Equation

**DOI:** 10.1371/journal.pone.0141068

**Published:** 2015-10-30

**Authors:** Evgenia S. Babushkina, Nikolay M. Bessonov, Fazoil I. Ataullakhanov, Mikhail A. Panteleev

**Affiliations:** 1 Institute for Problems of Mechanical Engineering, Russian Academy of Sciences, Saint Petersburg, Russia; 2 Department of Mathematics and Computer Science, Free University of Berlin, Berlin, Germany; 3 Department of Physics, Moscow State University University, Moscow, Russia; 4 Federal Research and Clinical Center of Pediatric Hematology, Oncology and Immunology, Moscow, Russia; 5 HemaCore LLC, Moscow, Russia; 6 Center for Theoretical Problems of Physico-Chemical Pharmacology, Russian Academy of Sciences, Moscow, Russia; 7 Department of Translational and Regenerative Medicine, Faculty of Biological and Medical Physics, Moscow Institute of Physics and Technology, Moscow, Russia; INSERM, FRANCE

## Abstract

In this study, we considered a continuous model of platelet thrombus growth in an arteriole. A special model describing the adhesion of platelets in terms of their concentration was derived. The applications of the derived model are not restricted to only describing arterial platelet thrombus formation; the model can also be applied to other similar adhesion processes. The model reproduces an auto-wave solution in the one-dimensional case; in the two-dimensional case, in which the surrounding flow is taken into account, the typical torch-like thrombus is reproduced. The thrombus shape and the growth velocity are determined by the model parameters. We demonstrate that the model captures the main properties of the thrombus growth behavior and provides us a better understanding of which mechanisms are important in the mechanical nature of the arterial thrombus growth.

## Introduction

Hemostasis is an essential physiological process that forms a platelet aggregate at the site of a blood vessel injury to stop bleeding. One of the major mechanisms of hemostasis is the adhesion of specialized blood cells, namely, platelets, to the damaged region and their aggregation to each other. This process forms a hemostatic plug that stops bleeding. In many pathological cases, the same mechanisms can lead to thrombosis—formation of an intra-vessel aggregate that occludes the vessel [1]. This mechanism of thrombosis is believed to be critical in the case of arterial flow [2]. The processes involved in arterial thrombus formation are numerous and complex [3]. Therefore, the development of a computational model that is capable of predicting the dynamics of platelet thrombus formation has significant bio-medical value.

The precise mathematical description of all the processes that are involved in hemostasis is a complicated task [4], and generally, simplifying assumptions are made such that the main features of the thrombus formation dynamics are still captured. In the following we assume that platelets are quickly activated after attachment to the vessel wall or to other platelets in a thrombus. This assumption allows us to neglect the difference between activated and non-activated platelets and bio-chemical reactions within the process. Based on this, we thus assume that the thrombus formation can be described as the adsorption of platelets to the vessel wall.

Earlier studies suggested different models of adsorption. The kinetics of the process is often described by adsorption reaction models in a number of studies [5]. These models are ordinary differential rate equations in solution systems. The adsorption diffusion models in [5] were developed to describe film diffusion and intraparticle diffusion in the process; thus, they can represent adsorption more accurately. However, none of these models allow the dynamics of adsorption to be modeled in space and time simultaneously, which is crucial for the thrombus formation phenomenon. Detailed models that combine modeling of thrombus growth and descriptions of biochemical reactions were suggested in [6, 7]. Although such models allow a comprehensive description of the process, it is not easy to perform a qualitative analysis and identify the key elements of the process. In this work, we propose a novel adsorption model that can be applied for simulation of thrombus formation.

## Model development and description

### Computational models of arterial thrombosis

Comprehensive reviews of computational modeling in the field of hemostasis and thrombosis can be found in [8–13]. Among such models, those addressing arterial thrombosis come in many varieties including analytical approximations of platelet adhesion and continuum models [14, 15], particle-based discrete models [16, 17], and fully three-dimensional simulations of platelet attachment with deformable cells [18, 19] that attempt to fully resolve the structure of flowing blood.

Each of these approaches has its specific advantages and shortcomings. Three-dimensional models have the most potential to fully grasp all intricacies of thrombus formation, but they cannot yet be used for this task: even with the help of supercomputers, such models require weeks to simulate several real-world seconds of a single experiment. Therefore, three-dimensional models, and even many two-dimensional ones, are predominantly used not for the simulation of thrombosis but rather for the analysis of single cell attachment [19]. Two-dimensional models without cell deformation can describe the entire process of thrombus growth [16]; however, they do not allow qualitative analysis of the adhesion, only numerical experiments.

Finally, the continuum-based models are an approximation that overlooks the fates of single platelets and that adresses platelet concentrations and mechanical properties of the thrombus medium. The main shortcomings of this approach include poor small-scale accuracy and the need to include phenomenological mechanical properties of a thrombus. However, these models allow analytical investigations of the thrombus formation laws and the development of theories. For this purpose, however, one would need to develop such a model in a way that would be suitable for analysis, i.e., in the form of simple reaction-diffusion equations with smooth functions or another analyzable form; complex rule-based continuum models suitable for computational simulations only [14] would not be sufficient. In the present study, we aimed at the development of a model to overcome this problem.

### 1D attachment of particles

**Fig 1:**
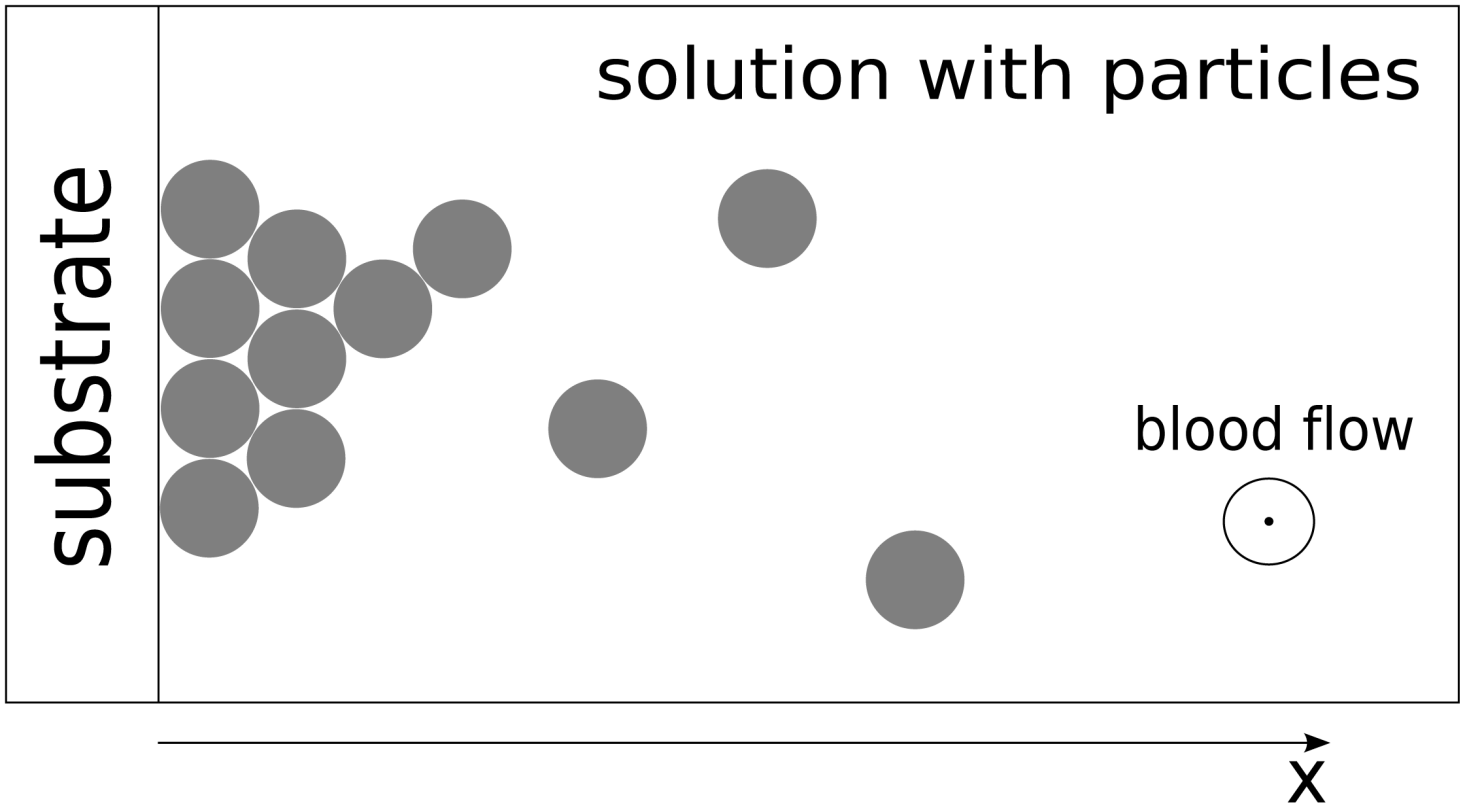
Multi-layer particle attachment to a substrate from a solution. The substrate (collagen or stable region of the thrombus) is on the left. Adhesion of particles from the suspension leads to an increase of the particle aggregate on the surface of the substrate such that it grows to the right.

To obtain a continuous model describing thrombus development, let us first consider the one-dimensional attachment of suspended particles to each other, as depicted in Fig 1. This figure is a representation of a region at the boundary of a thrombus, which is sufficiently small such that we can assume the local rate of platelet adhesion (in general depending on the local hematocrit and shear rate of the fluid) to be constant throughout this region. In other words, we locally approximate platelet attachment using a “chemical adhesion” model [20]. This approximation in the 1D case does not take into account platelet re-arrangement and rolling along the thrombus (which will be added later for the 2D case). For simplicity, in the initial derivation, we will only consider irreversible attachment.

Let us denote the mean concentration of adhered particles in the aggregate with the variable *C*. Because we consider the one-dimensional case here, we assume that the thrombus can only grow in the direction *x* (see Fig 1); however, the concentration *C* of the particles at each value of *x* can vary from 0 to *C*_*max*_, where *C*_*max*_ corresponds to the situation in which the channel is fully packed by the particles in the vertical direction.

Although real platelets are distributed non-uniformly in the vessel, we are only interested in their concentration in the region close to the thrombus. Therefore, we assume that the volume concentration of the un-attached particles in the suspension is constant, *C*_*na*_. Let us now consider the evolution of the thrombus within a small time interval Δ*t*. The concentration of the adhered platelets *C* at each spatial point along the *x* axis over this time interval increases according to the following rules:

1. It is proportional to the mean concentration of the non-attached particles. In time, the mean concentration *C*_*na*_ changes, because some fraction of the volume (*C*/*C*_*max*_) is occupied by the already aggregated particles. Therefore, the mean concentration of the non-aggregated platelets is *C*_*na*_(1 − *C*/*C*_*max*_); i.e., it is zero where an aggregate is formed and *C*_*na*_ everywhere else.
2. It is proportional to the length of the time interval Δ*t*.
3. There is a proportionality coefficient or apparent rate constant *k*_*eff*_ (determined by local hematocrit, the shear rate, and intrinsic molecular characteristics of the attachment reactions; furthermore, we will also include the particle concentration *C*_*na*_ in this constant).
4. The overall process is in essence similar to a two-species reaction; it is proportional to the concentration of free attachment sites provided by particles that have already adhered. This will be illustrated below.

**Fig 2:**
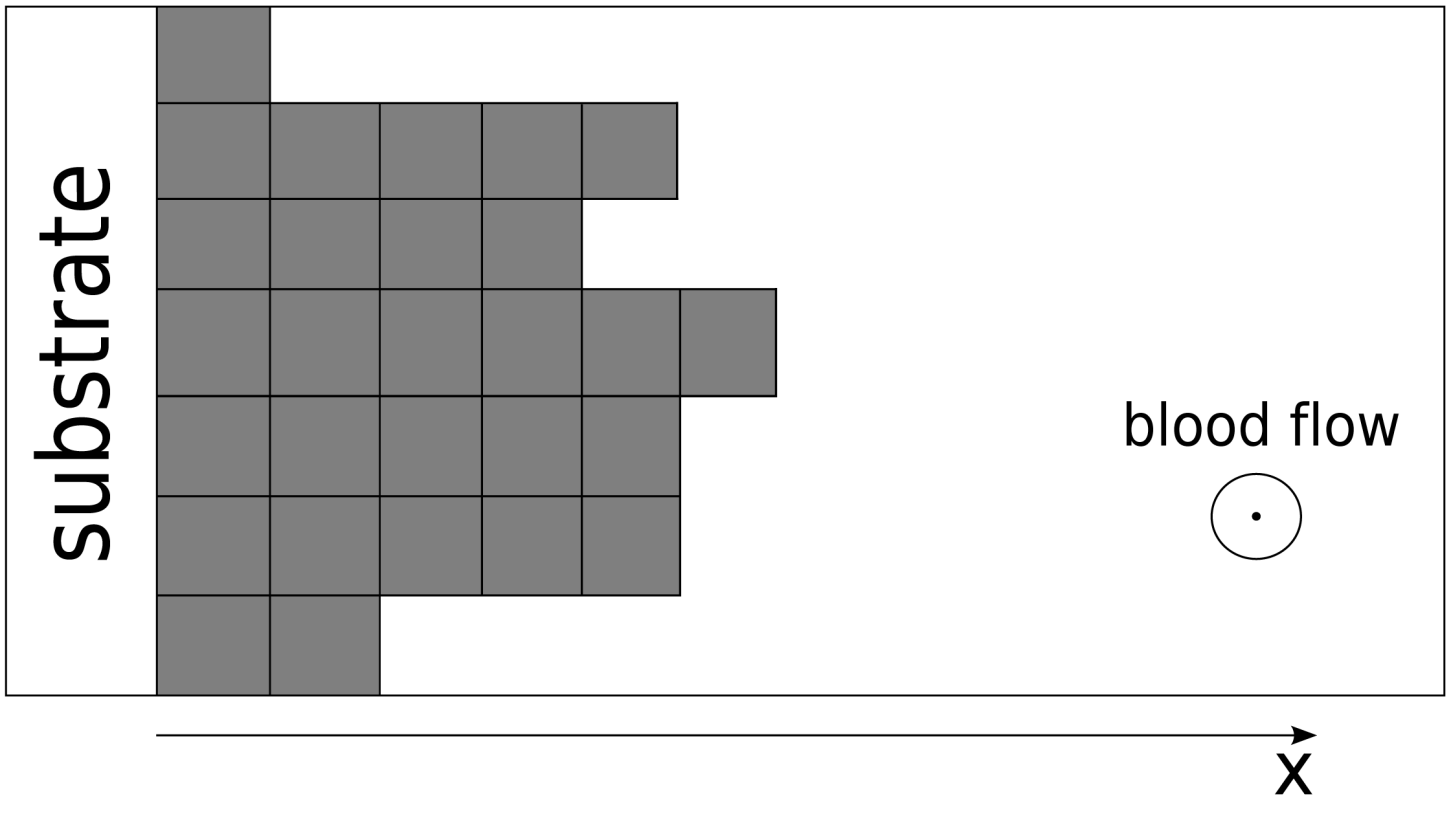
Multi-layer particle attachment re-drawn to illustrate individual layers. In all aspects, this Figure illustrates the same mechanism as Fig 1.

Let us analyze the structure of the binding sites in more detail. For a better understanding, let us replace the concentration of platelets by the number of particles *N* and the concentration of free sites by the number of these sites. We can easily exchange these quantities, because they have the same nature. We also represent attaching particles as rectangles and redraw Fig 1 in an orderly fashion, displaying layers as depicted in Fig 2.

**Fig 3:**
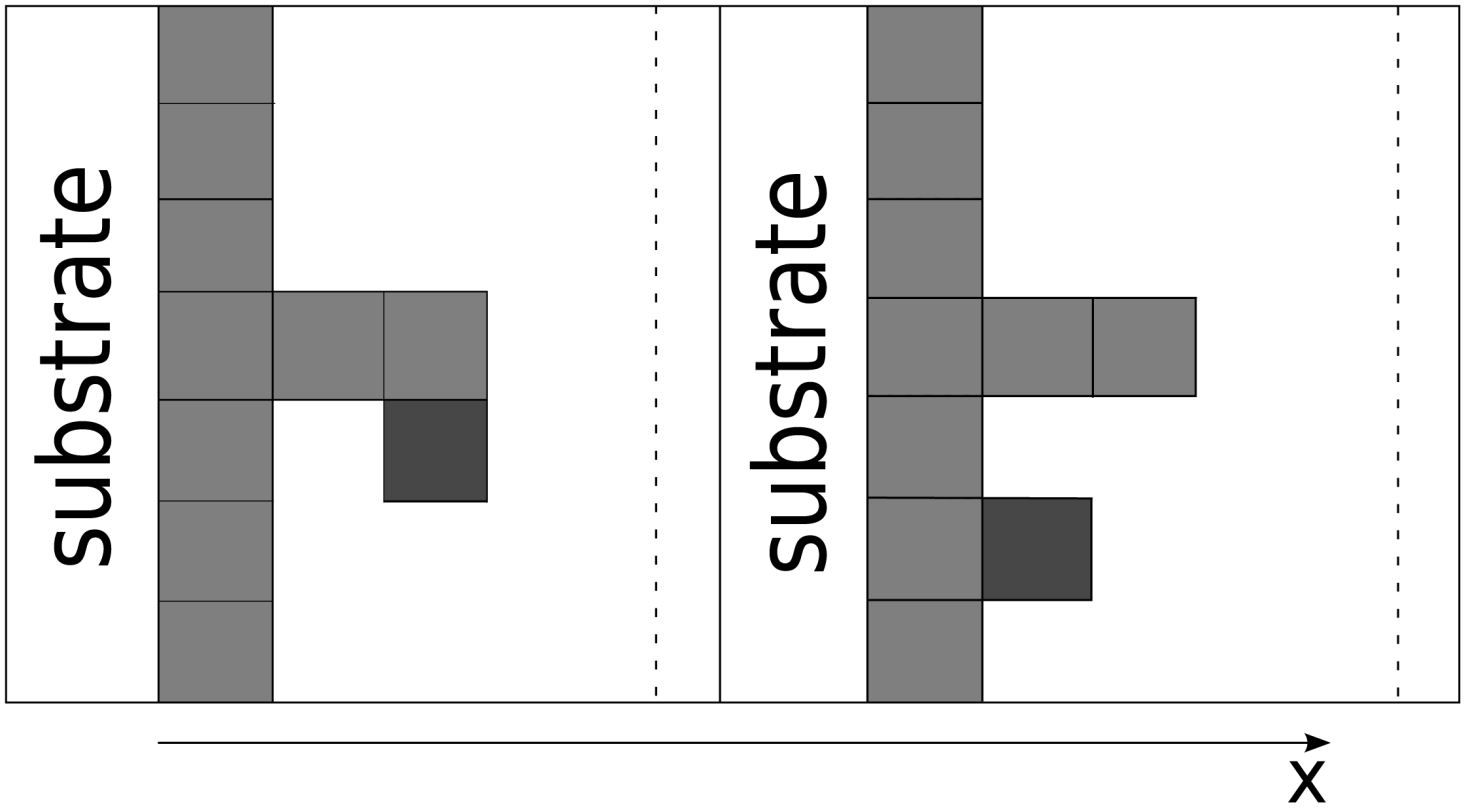
Two types of particle attachment. One is determined by the ‘free space’ in the aggregate, and the other depends on the number of ‘free ends’.

The particles arrive from the solution and form layer upon layer in a manner not dissimilar to the Tetris game. There is no requirement for a layer to be completed when the following layer begins to be built. Fig 3 clearly illustrates the fact that there are two types of binding sites for particle attachment. The first type is provided by the ‘side planes’ of each attached rectangle. Attachment to these sites does not increase the local aggregate height but rather increases its density by ‘filling out’ cavities on the thrombus surface. The second type is provided by the ‘free ends’ of each column, and this type of attachment provides a possibility of initiating a new layer. This is the mechanism that actually propagates a thrombus in space. These two types of particle attachment are schematically depicted in Fig 3. In reality, there is no such clear distinction because real particles or platelets are not square bricks; nevertheless, these two types of attachments are present.

Let us now consider a small interval Δ*x* along the *x* axis (from *x* to *x* + Δ*x*) and determine the increase in the number of attached platelets in this interval within the time interval Δ*t* (from *t* to *t* + Δ*t*).

Let us consider the second type of particle attachment (Fig 3 left). The number of ‘free ends’ can be defined as the change of *N* within the interval Δ*x*, i.e., ∣*N*(*x*) − *N*(*x* + Δ*x*)∣. Next, we consider the first type of attachment. The number of ‘side planes’ available for attachment can be defined as the average (arithmetic average) number of particles corresponding to the columns in the interval multiplied by the number of layers within the interval [*x*, *x* + Δ*x*], which equals Δ*x*/*λ*, where *λ* is the characteristic size of the particle, i.e., *N*_*av*_Δ*x*/*λ*. Uncertainty of the geometric model might change this several-fold with no effect on the overall validity; this can be corrected by adjustment of *λ*.

All the above reasoning remains the same for the concentrations of platelets and ‘free attachment sites’. Therefore, taking into account that we considered a small interval Δ*x*, we obtain:

$$\begin{array}{c} {C|}_{t+\Delta t}{-C|}_{t}∼\frac{C}{\lambda }+\left|\frac{dC}{dx}\right|. \end{array}$$ (1)

By combining assumptions 1–3 and model Eq (1), we arrive at the equation of thrombus propagation:

$$\begin{array}{c} \frac{\partial C}{\partial t}=k_{eff}\left(1-\frac{C}{C_{max}}\right)(\frac{C}{\lambda }+|\frac{\partial C}{\partial x}|), \end{array}$$ (2)

where *C*_*max*_ is the maximum value of the concentration and *k*_*eff*_ is the coefficient of platelet adhesion efficiency. After dividing both parts of the equation by *C*_*max*_ we can obtain the dimensionless form of the equation and denote the now unitless concentration varying within the range [0, 1] again by *C*.

### Analysis of the platelet adhesion equation

Note that Eq (2) is not completely novel in the field of adsorption models. Similar models have already been obtained based on the Brunauer-Emmett-Teller (BET) adsorption theory [20]. These types of models include the first spatial derivative of the concentration of the adhered particles (because adsorption is determined by the ‘fractional surface’ of each layer) and do not have a diffusion term (because adsorbed material does not diffuse). However, this theory assumes the presence of a single binding site provided by each particle for the formation of the uppermost layer. In our case, this would lead to a simplified (but more obvious) model, where the increase of particle concentration only depends on the concentration gradient:

$$\begin{array}{c} \frac{\partial C}{\partial t}=k_{eff}\left(1-\frac{C}{C_{max}}\right)\left(-\frac{\partial C}{\partial x}\right). \end{array}$$ (3)

Note that the term $\frac{\partial C}{\partial x}$ is always negative in the 1D case if the concentration propagates in the positive direction of *x*; therefore, the equation can also be rewritten in a form similar to Eq (2):

$$\begin{array}{c} \frac{\partial C}{\partial t}=k_{eff}\left(1-\frac{C}{C_{max}}\right)\left|\frac{\partial C}{\partial x}\right|. \end{array}$$ (4)

The latter form is more convenient because we want to extend the model to the 2D case.

The simplest model similar to that in the present study is the one by Rosen [21] that does not consider the saturation of binding sites:

$$\begin{array}{c} \frac{\partial C}{\partial t}=k_{eff}\left(-\frac{\partial C}{\partial x}\right). \end{array}$$ (5)

Regardless, most studies based on the BET theory for gases do not use partial differential equations but rather focus on ordinary equations assuming equilibrium of the outermost layer. Removal of these two limitations constitutes the main difference between our approach and the previous approaches.

### Solution of the 1D model

Eq (2) describes the spatial propagation of a one-dimensional platelet thrombus. To characterize and evaluate the dynamics of the thrombus growth, we introduce the following quantity that shows how fast the concentration is changing in the direction normal to the wall of the channel; we call this quantity velocity.

$$\begin{array}{c} V=\frac{d\int_{0}^{L}C\left(x\right)dx}{dt}, \end{array}$$ (6)

where *L* is the length of the computational domain.

**Fig 4:**
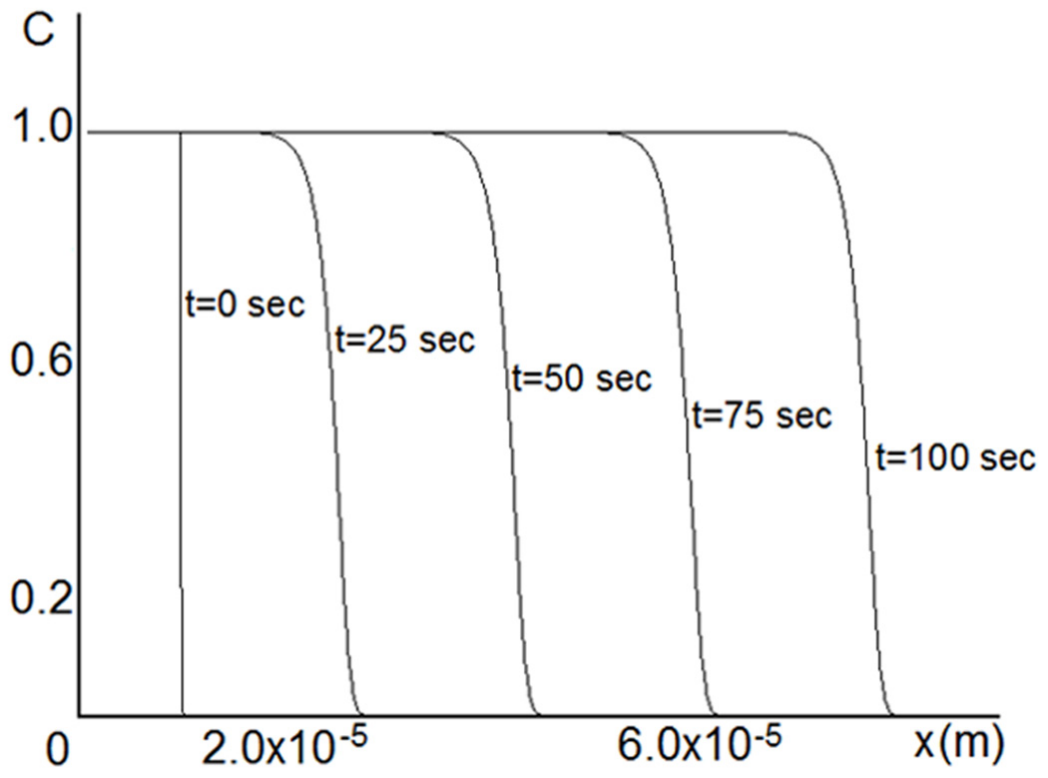
Numerical solution of Eq (2). Propagation of a traveling-wave solution of Eq (2) for *λ* = 1 ⋅ 10^−6^ *m* and *k*_*eff*_ = 5 ⋅ 10^−7^ *m*/*sec*. Concentration increases in time in the positive direction of *x*.

The velocity and the profile of the wave depend on the model parameters. The equation was solved numerically on a uniform mesh using an explicit finite difference method. The solution resembles a traveling wave (Fig 4), although the equation does not have a diffusion term and is not a typical chemical traveling wave in the true sense of the word. The simulation was performed for the following parameter values: *λ* = 10^−6^ *m*, *k*_*eff*_ = 5 ⋅ 10^−7^ *m*/*sec*.

**Fig 5:**
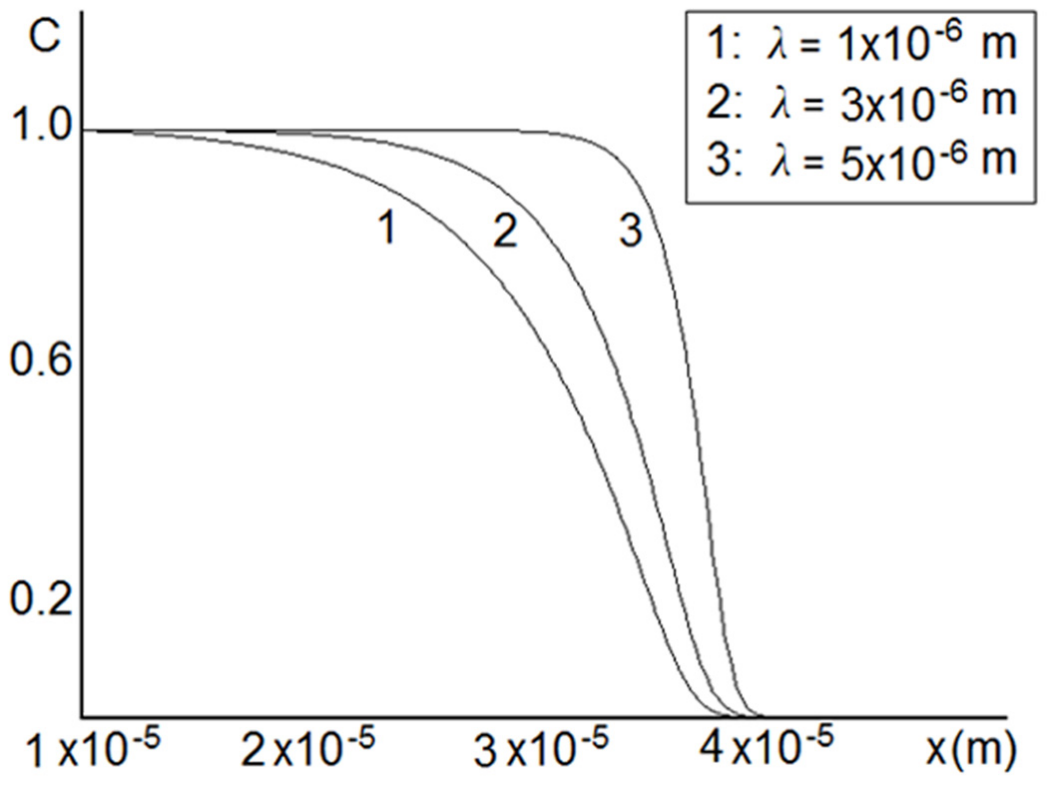
Numerical solution of Eq (2). Dependence of concentration profiles on the parameter *λ* for *k*_*eff*_ = 5 ⋅ 10^−7^ *m*/*sec*. The steepness of the concentration profiles increases with increasing *k*_*eff*_.

**Fig 6:**
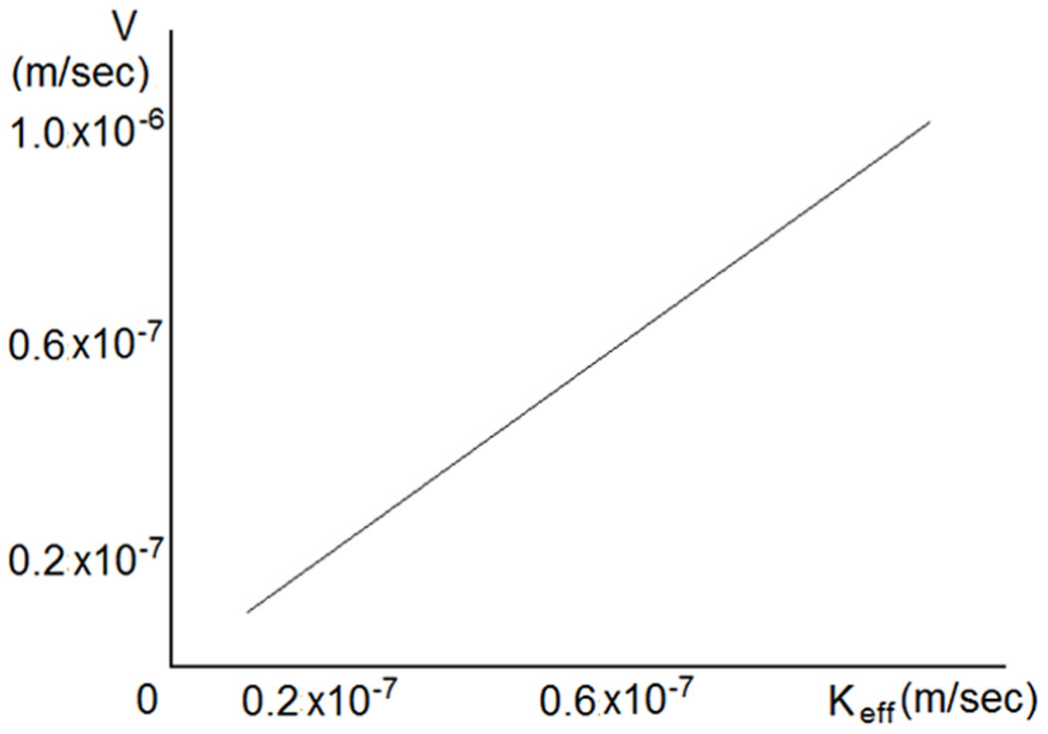
Numerical solution of Eq (2). Dependence of wave propagation velocity on the parameter *k*_*eff*_ for *λ* = 1 ⋅ 10^−6^ *m*. Coefficient *k*_*eff*_ controls the velocity of wave propagation.

As shown in Fig 5, the steepness of the concentration profile depends on the coefficient *λ*: it increases with increasing *λ*. Regarding the velocity of the wave propagation, Fig 6 shows that there is a linear dependence of the velocity on the coefficient *k*_*eff*_.

### 2D case

Because the behavior of a platelet clot in the blood flow strongly depends on the flow properties, we propose a two-dimensional model that combines the blood flow and the platelet clot dynamics description.

$$\begin{array}{c} \frac{\partial C}{\partial t}=k_{adh}\gamma \left(1-\frac{C}{C_{max}}\right)(\frac{C}{\lambda }+\left|\nabla C|\right)-k_{rol}\nabla \cdot \left(\bar{v}C\right), \end{array}$$ (7)

where $\bar{v}$ is the blood velocity, $\gamma =\gamma (\bar{v})$ is the wall shear rate of the fluid, *k*_*adh*_ is the coefficient of platelet adhesion efficiency, and *k*_*rol*_ is the coefficient of platelet movability within a thrombus. The second term on the right-hand side of the equation models the convection. By introducing this term, we allow the concentration of the platelets to be adjusted by the flow and the intensity of such adjustment is defined by the constant *k*_*rol*_. We assume that the movement of platelets by the flow is one of the key processes in the thrombus formation. We further assume that the concentration propagation depends on the wall shear rate *γ*, and as we do not assume it to be constant in the two-dimensional case, *k*_*eff*_ is replaced by *k*_*adh*_ *γ*.

**Fig 7:**
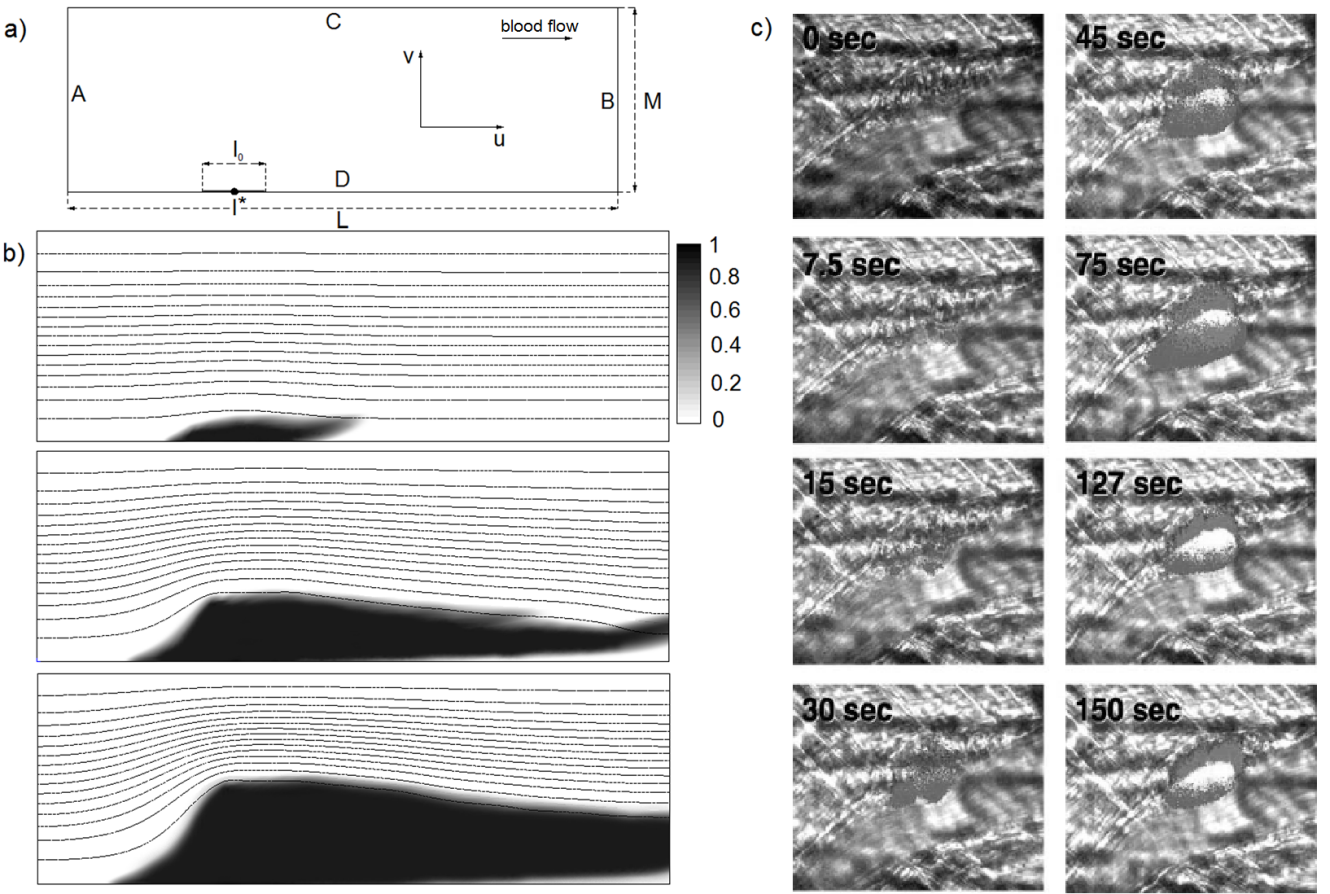
2D model. a) Scheme of computational domain for the two-dimensional problem used for all simulations; b) an example of the thrombus dynamics for *λ* = 3 ⋅ 10^−6^ *m*, *k*_*adh*_ = 8 ⋅ 10^−11^ *m*, *k*_*rol*_ = 7 ⋅ 10^−5^, *C*_*B*_ = 0.9 and *l*_0_ = 15 ⋅ 10^−6^ *m*; the thrombus is growing in time according to Eq (7); and c) developing thrombus of a living wild-type mouse following endothelial injury. Blood flow is from right to left. The time, in seconds, indicates the approximate interval from vessel wall injury to image capture. From: Celi A, Merrill-Skoloff G, Gross P, Falati S, Sim S, Flaumenhaft R, Furie BC, Furie B. Thrombus formation: direct real-time observation and digital analysis of thrombus assembly in a living mouse by confocal and widefield intravital microscopy. Journal of Thrombosis and Haemostasis. 2003; 1: 60–68. Retrieved 19 July, 2015, from John Wiley and Sons.

We consider the blood flow in a two-dimensional rectangular domain (see Fig 7a) with height *M* = 5 ⋅ 10^−5^ *m* and length *L* = 15 ⋅ 10^−5^ *m*. The following boundary conditions were chosen for Eq (7):

$$\begin{array}{c} {C|}_{A}=0,\frac{\partial C}{\partial x}{|}_{B}=0,\frac{\partial C}{\partial y}{|}_{C,D}=0. \end{array}$$ (8)

As an initial condition for Eq (7) we set the platelet concentration to be equal to 1 on a part of the boundary D of length *l*_0_ (Fig 7a), which imitates an injury on the vessel wall.

We assume that blood plasma is a Newtonian incompressible homogeneous fluid with constant viscosity, which can be modeled using the Navier-Stokes equations in the following form:

$$\begin{array}{c} \frac{\partial \bar{v}}{\partial t}+\left(\bar{v}\cdot \nabla \right)\bar{v}=\nu \Delta \bar{v}-\frac{1}{\rho }\nabla p, \end{array}$$ (9)

$$\begin{array}{c} \nabla \cdot \bar{v}=0, \end{array}$$ (10)

where $\bar{v}=(u,v)$ is the flow velocity, *p* is the pressure, *ν* is the viscosity of the fluid, and *ρ* is the density of blood.

Eqs (9) and (10) are supplemented by no-slip boundary conditions on the boundaries C and D (see Fig 7a), and there are two options for the boundary conditions on sides A and B: pressure gradient or velocity profiles.

To couple Eqs (7), (9) and (10), we set the boundary D to be varying in time and define an additional parameter of the model *C*_*B*_, which determines a new boundary for the flow. If the value of the concentration *C* at some point in the domain (*x*′, *y*′) reaches this value, i.e., *C*(*x*′, *y*′) = *C*_*B*_, this point becomes a part of the new boundary D, i.e., $\bar{v}(x^{\prime },y^{\prime })=0$. This particularly implies that there is no fluid flow inside the thrombus, i.e., that the thrombus is a rigid obstacle for the fluid; the fluid can penetrate the thrombus only in those areas where the concentration is less than *C*_*B*_.

The two-dimensional problem was also solved numerically on an orthogonal uniform mesh. The explicit finite difference method was used for the numerical solution of Eq (7). The projection method introduced by Chorin [22] was used to solve the Navier-Stokes Eqs (9) and (10). For all simulations, we set *ρ* = 1060*kg*/*m* ^3^ and *ν* = 5 ⋅ 10^−6^ *m* ^2^/*sec*, which corresponds to the parameters of real blood. We further set the value Δ*p* = 30*Pa* in the case of pressure gradient boundary conditions or the wall shear rate equal to 1000 1/*sec* for the velocity profile boundary conditions. These values create approximately equivalent velocity fields at the initial moment of time and correspond to the conditions of real blood.

### Model assumptions and limitations

The model developed above is designed specifically for arterial thrombus formation caused by platelet adhesion to the damaged vessel wall. This model is continuous; thus, it is not applicable at characteristic spatial scales comparable to the size of a platelet. It does not consider fibrin formation because the overall dynamics of such a thrombus is defined by the “shell” of weakly activated, discoid platelets with little fibrin at the core ([23], see also the analysis in [24]). Therefore, the model cannot be applied to venous thrombosis. The model does not explicitly include platelet activation, although it is assumed to occur upon platelet attachment. There is no explicit detachment of platelets, although it is assumed to affect the overall adhesion constant.

We assumed the platelet concentration to be uniformly distributed along the vessel width, which is only a rough approximation because it is known that platelet concentration increases near vessel walls; however, the concentration near the wall is the only concentration of importance for thrombus formation. In future works, the fluid flowing inside the thrombus according to the Darcy’s law could be considered. In the current work, we neglected the fluid flow inside the thrombus (because the velocities are expected to be small) and approximated the flow at the boundary of the thrombus using the Navier-Stokes equations.

## Results

An example of the thrombus growth dynamics is shown in Fig 7b. The platelet concentration field is indicated by different shades of black, and stream lines of the fluid are shown in the pictures. This simulation was conducted for the pressure gradient boundary conditions and the following parameter values: *λ* = 3 ⋅ 10^−6^ *m*, *k*_*adg*_ = 8 ⋅ 10^−11^ *m*, *k*_*rol*_ = 7 ⋅ 10^−5^ and *C*_*B*_ = 0.9, *l*_0_ = 15 ⋅ 10^−6^ *m*. The torch-like shapes of the obtained thrombus are qualitatively similar to the shapes of a developing thrombus in a living wild-type mouse following endothelial injury experimentally obtained in [25] (see Fig 7c).

**Fig 8:**
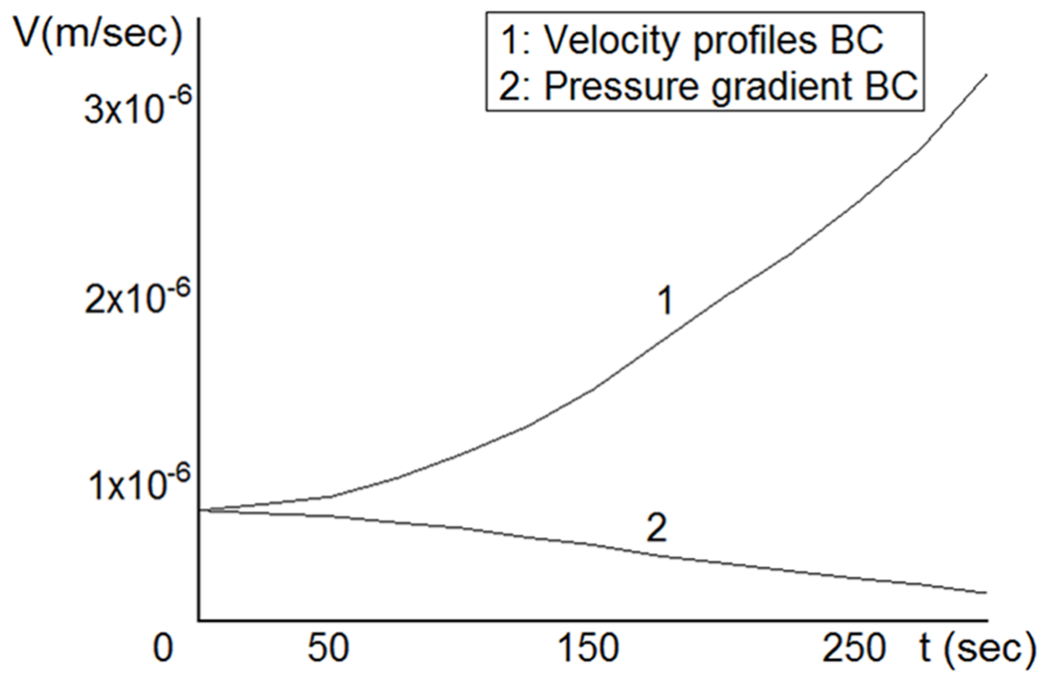
2D model. Dependence of the wave velocity in time on the boundary conditions; the pressure gradient boundary condition gives a realistic behavior, as the thrombus expansion slows down in time.

It can be observed that the behavior of the thrombus in the blood flow depends on the boundary conditions on sides *A* and *B* for the blood flow. The dependence of the wave velocity on the boundary conditions in time is shown in Fig 8. The wave velocity is defined as

$$\begin{array}{c} V=\frac{d\int_{0}^{M}{C\left(x,y\right)dy|}_{x=l^{*}}}{dt}, \end{array}$$ (11)

where *M* is the height of the computational domain and l* corresponds to the middle point of the initial condition for the concentration (see Fig 7a). Fig 8 shows that the velocity profile boundary conditions yield an increase in the wave velocity that does not allow stopping of the thrombus growth. The shear rate of the fluid next to the thrombus is monotonically increasing, which makes the thrombus growth increasingly more intensive. This does not correspond to the real thrombus dynamics. We therefore conclude that the pressure gradient is a natural boundary condition for the blood flow simulation. For all simulations, we chose the pressure gradient boundary conditions because they were more realistic.

**Fig 9:**
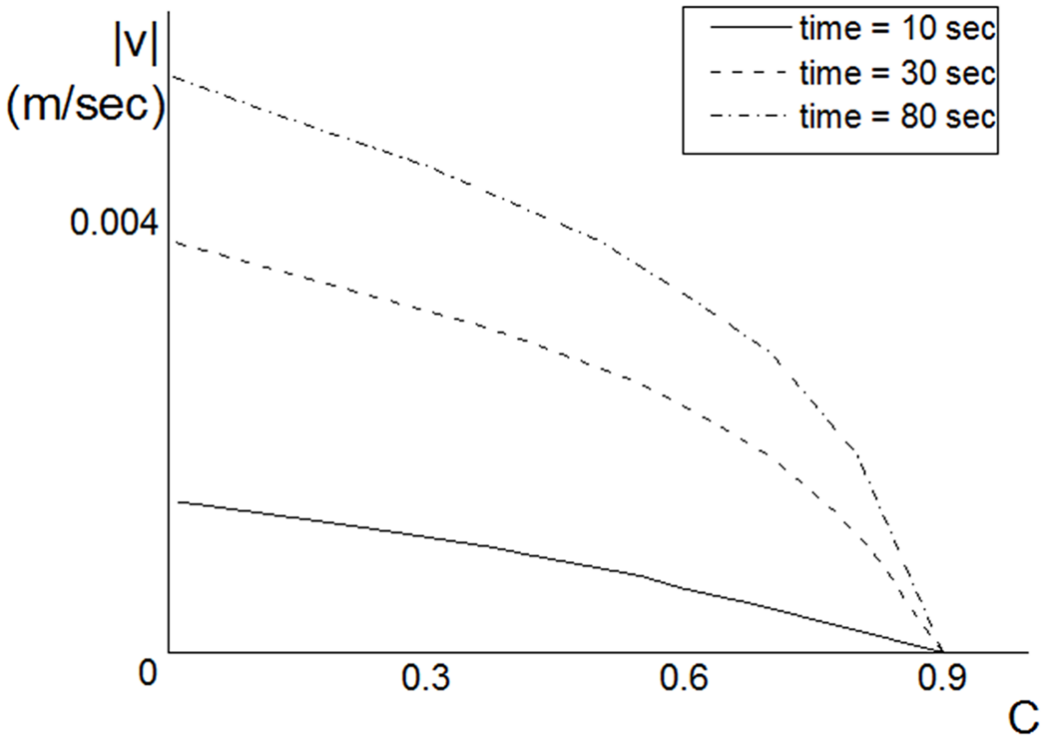
Dependence of the fluid velocities on the concentration for the following parameter values: *λ* = 3 ⋅ 10^−6^ *m*, *k*_*adh*_ = 8 ⋅ 10^−11^ *m*, *k*_*rol*_ = 7 ⋅ 10^−5^, *C*_*B*_ = 0.9 and *l*_0_ = 15 ⋅ 10^−6^ *m* and the pressure gradient boundary condition. Velocities increase with the decreasing concentration values.

Fig 9 shows the dependence of the fluid velocity on the concentration values. According to our model, the fluid may penetrate the thrombus only in those areas where *C* < *C*_*B*_; i.e., we do not consider convection of concentration within the thrombus, only at its boundary. As expected, the velocities increase with decreasing concentration, but they remain relatively small.

**Fig 10:**
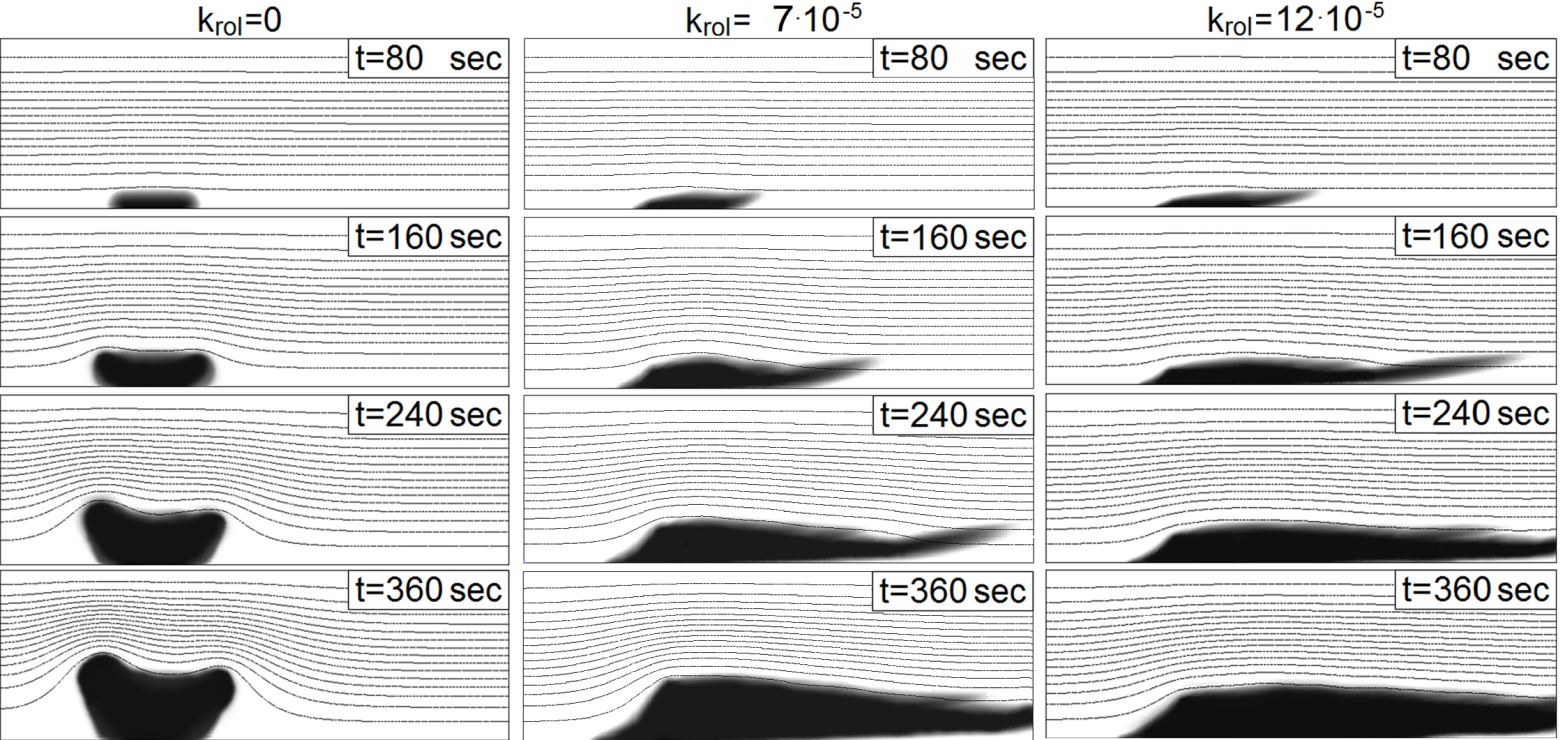
Behavior of a thrombus in time regarding the dependence on the coefficient *k*_*rol*_ for *λ* = 3 ⋅ 10^−6^ *m*, *k*_*adg*_ = 8 ⋅ 10^−11^ *m*, *C*_*B*_ = 0.9 and *l*_0_ = 15 ⋅ 10^−6^ *m*. Coefficient *k*_*rol*_ controls the convection term in Eq (7); the larger the value of the coefficient is, the more the thrombus is moved by the fluid.

Our simulations further show that the convective term in Eq (7) plays an essential role. The behavior of the thrombus without convective transport of platelets within the thrombus (*k*_*rol*_ = 0) and with it is shown in Fig 10. The dynamics of the thrombus with *k*_*rol*_ = 0 would be unstable under realistic conditions [23], whereas accounting for the convective term allows obtaining quite realistic torch-like shapes of a thrombus in the flow. However, note that the behavior (and the shape) of the thrombus depends on the choice of the parameters of the model. If the first term on the right-hand side of Eq (7) dominates over the second term, we can expect the thrombus to be growing in the direction normal to the channel wall. By contrast, if convection is strong, the thrombus can develop only in the direction of the flow. There is also a third possibility, i.e., the two terms are in equilibrium, which would mean that the thrombus stops any development in the direction normal to the channel wall. Different types of behaviors can also change one another, because the system is complex and dynamic. The realistic behavior consists of growing during the first stage and equilibrium in the second stage.

**Fig 11:**
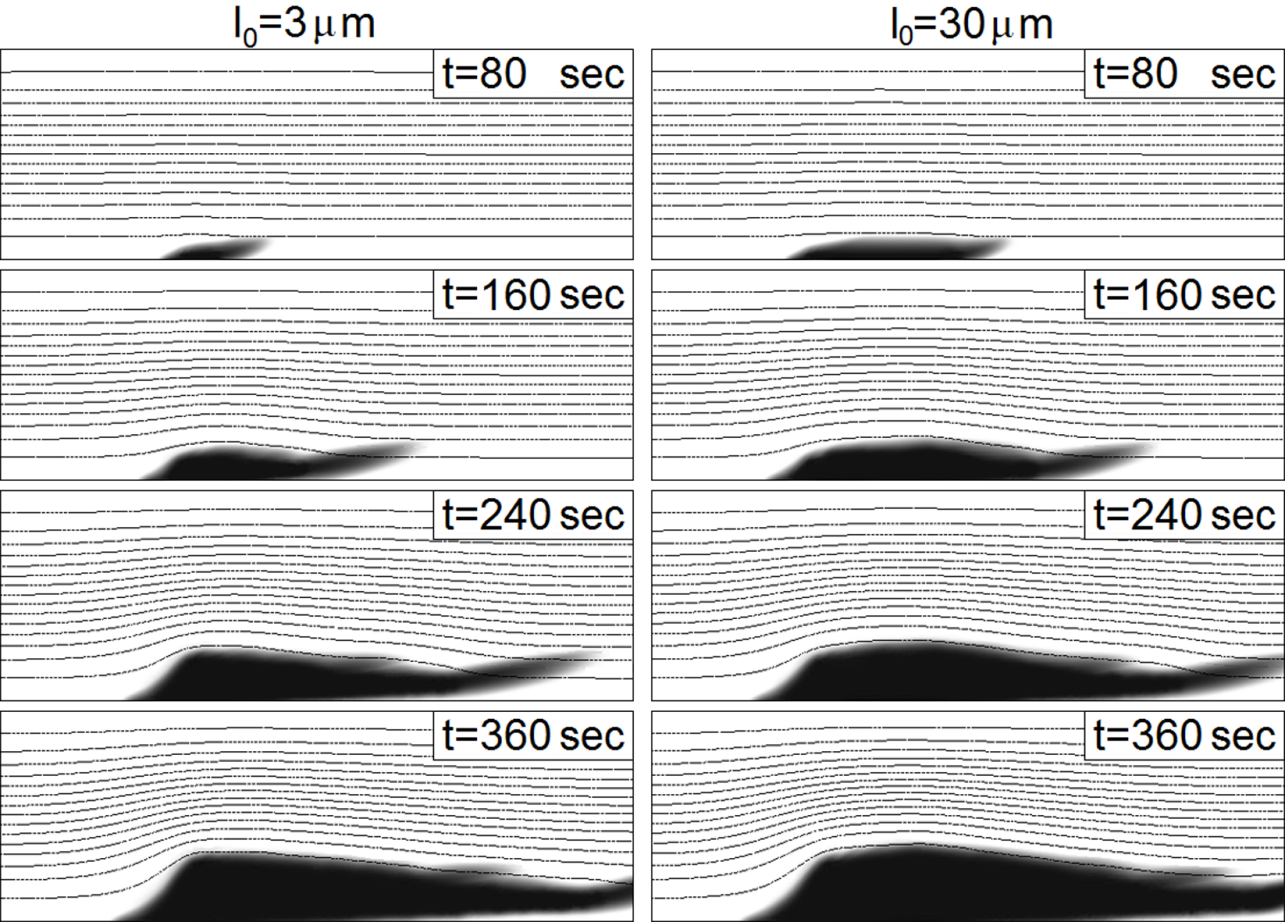
Behavior of a thrombus in time regarding the dependence on the length of an injury for *λ* = 3 ⋅ 10^−6^ *m*, *k*_*adg*_ = 8 ⋅ 10^−11^ *m*, *k*_*rol*_ = 7 ⋅ 10^−5^ and *C*_*B*_ = 0.9. The length of the injury does not considerably influence the behavior.

Finally, we also observed in our simulations a weak dependency of the final size of the thrombus on the size of the injury (see Fig 11). As shown in this figure, a 10-fold difference in the initial size of the injury leads to a very small difference in the size in the later stages of the thrombus development.

## Discussion

In this paper, we derived and investigated a new continuous adsorption model that can be used for the prediction of arterial platelet thrombus formation in flowing blood. The developed model is concise, laconic and able to capture all critical features of the process.

The major advantage of this model compared with the adsorption models reported previously [5, 26–30] is that it includes spatial heterogeneity and also considers growth of the surface due to absorbed material in the explicit form. Although this feature is critical for thrombus growth, this model can also be of importance for many other fields, such as physics, chemistry, and biology.

The arterial thrombus growth pattern was investigated as a function of the model parameters. The one-dimensional model provides a curious traveling excitation wave solution, which (in contrast to traditional excitation waves [31, 32]) does not require a diffusion term. In the two-dimensional case, it was found that it is crucial to take into account the movement of the adhering platelets at the boundary of the thrombus by the blood flow; otherwise, any adhesion model would result in thrombus growth opposing the flow. Using our model, we obtained a realistic torch-like shape of the thrombus in the flow, which is qualitatively similar to the shapes obtained experimentally [23, 25]. Finally, we observed only a weak dependence of the thrombus dynamics on the injury size; it would be interesting to confirm this result through experimental studies.
